# Estimating the copepod biomass in the North West African upwelling system using a bi-frequency acoustic approach

**DOI:** 10.1371/journal.pone.0308083

**Published:** 2024-09-06

**Authors:** Ndague DIOGOUL, Patrice BREHMER, Rainer KIKO, Yannick PERROT, Anne Lebourges-Dhaussy, Elizandro RODRIGUES, Abou THIAM, Anne MOUGET, Salaheddine EL AYOUBI, Abdoulaye SARRÉ

**Affiliations:** 1 IRD, CNRS, Ifremer, Lemar, SRFC, CSRP, University Brest, Dakar, Senegal; 2 IRD, CNRS, Ifremer, Lemar, DR Ouest, University Brest, Plouzané, France; 3 Institut Sénégalais de Recherches Agricoles, ISRA, Centre de Recherches Océanographiques de Dakar Thiaroye, CRODT, Dakar, Senegal; 4 Laboratoire d’Océanographie de Villefranche (LOV), Institut de la Mer de Villefranche (MEV), Villefranche-sur-Mer, France; 5 GEOMAR Helmholtz Centre for Ocean Research Kiel, Kiel, Germany; 6 Instituto del Mar, IMar, Mindelo, Island of Sao Vicente, Cabo Verde; 7 Institute of Environmental Science (ISE), University Cheikh Anta Diop UCAD, Dakar, Senegal; 8 Institut National de Recherche Halieutique, INRH, Agadir, Morocco; University of Connecticut, UNITED STATES OF AMERICA

## Abstract

The Canary Current Large Marine Ecosystem (CCLME) is one of the most productive Large Marine Ecosystems worldwide. Assessing the abundance, biomass and distribution of zooplankton in the southern part of this system, off the coast of West Africa, remains challenging due to limited sampling efforts and data availability. However, zooplankton is of primary importance for pelagic ecosystem functioning. We applied an inversion method with combined analysis of acoustic and biological data for copepod discrimination using a bi-frequency (38 and 120 kHz) approach. Large copepods with equivalent spherical radii > 0.5 mm were identified using differences in the mean volume backscattering strength (MVBS). Regarding abundance measured by net sampling, copepods strongly dominated the zooplankton community and the large fraction account for 18%. This estimate correlated significantly with MVBS values that were obtained using an inverse algorithm. We confirmed the utility of using 38 kHz for large copepod detection. An epipelagic biomass of large copepod was estimated at 120–850 mg m^-2^ in March during upwelling season. It is worth noting that this estimation likely underestimates the true biomass due to inherent uncertainties associated with the measurement method. We recommend future investigations in the interest of using only nighttime data to improve the sampling pattern, particularly on the upper part of the water column (< 10 m) as well as on the shallow part of the continental shelf (< 20 m depth) not covered by fisheries vessel. Nevertheless, such high copepod biomass supports high fish production underlining the key role of copepod in the CCLME. Our results open the way to the analysis of the fluctuation and trend of copepod biomass, along with three decades of fisheries acoustics data available in the region. This helps to determine ecosystem changes, particularly under climate change, and to investigate the role of copepods in the southern CCLME carbon pump at the fine scale.

## 1. Introduction

Zooplankton species are one of the most important biotic components of marine ecosystems. Zooplankton communities comprise a diverse assemblage of taxonomic groups that play varying but integral roles in marine food webs and biogeochemical cycles [1]. Among the most ecologically important are copepods, a subclass of tiny crustaceans that are predominant grazers of phytoplankton [2] and a crucial link transferring energy to higher trophic levels as prey for fish, mammals, and seabirds [3].

The order Calanoida typically dominates copepod abundance and biomass across many ocean regions [4]. Some calanoid families, like the Eucalanidae, can form dense surface swarms that are pivotal in marine food chains [5, 6]. Besides copepods, other major components of zooplankton include gelatinous groups like thaliaceans (salps, doliolids) and cnidarians (jellyfish, siphonophores), which can be efficient grazers but also act as zooplanktivores [7, 8]. The crustacean class also contains larger filter-feeding groups like euphausiids (krill) that are key prey for marine predators [9].

Knowledge of zooplankton abundance and biomass at various spatial and temporal scales remains a key element of marine ecosystem research [10]. Information on prey biomass is needed to study the relationship between larval fish feeding and food supply [11]. In Northwest African waters, copepods are the most common and abundant zooplanktonic group and play a major role in the marine food web. Their small size and high abundance make them a vital food source for fish larvae, juvenile fish, and many other marine organisms [12, 13]. The Canary Current Large Marine Ecosystem (CCLME) is an upwelling system which supports high marine productivity [14, 15]. An upwelling is a physical oceanographic process in which deep, rich water rises toward the surface [15, 16], promoting the growth of phytoplankton, a primary food source for copepods [17]. Consequently, increased phytoplankton production due to upwelling leads to higher food availability, resulting in increased copepod abundance and then marine biomass in areas affected by upwelling.

In the CCLME, copepods likely represent the principal prey item sustaining the early life stages of numerous commercially exploited small pelagic fish stocks of key economic value [18, 19]. Small pelagic fish constitute the bulk of the landings in the CCLME, with annual catches of the main species ranging from approximately 1.7 to 2.5 million tons over the last few decades [20]. The fisheries in this region, considered a multi-usage resource [21], support approximately one million jobs and serve as the primary source of livelihood for approximately 150,000 artisanal fishers [22].

Given their role as the predominant grazers and primary trophic pathway facilitating energy transfer to higher consumers, fluctuations in copepod population abundance and spatial patchiness affect fish recruitment variability, juvenile survival rates, and consequently, the annual productivity of pelagic fisheries [23, 24]. Information on copepod abundance, biomass and spatial distribution within this highly productive marine ecosystem, therefore, becomes a prerequisite for the reliable implementation of ecosystem-based fisheries management frameworks [25]. Quantifying copepods and mapping their spatial distribution could elucidate the bottom-up trophic controls that potentially drive the observed inter-annual fluctuations in the pelagic fish populations sustaining the national coastal fisheries [26]. Direct counts and measurements of organisms under the microscope, and precise determinations of mass, density, volume, etc., are generally difficult to obtain, tedious, and time-consuming [27]. Ideally, zooplankton biomass should be estimated from fresh material, but the challenges hinder direct biomass measurements in sorting biological material from net sampling [28]. Treating zooplankton samples is laborious and time-consuming, often resulting in measurements conducted on frozen or formalin-preserved specimens. Both methods introduce error and uncertainty [29, 30]. However, the main error source in biomass estimate from net sampling is likely attributed to their distribution patchiness vs sampling pattern, i.e. on a limited number of stations vs spatial coverage, the case with the acoustic method. Fisheries acoustics devices are among the most effective tools for the detection and mapping of organisms in the water column [31, 32], including zooplankton [33–35]. Acoustic backscattering techniques provide higher-resolution results than traditional net sampling strategies. However, the contributions of individual species are difficult to discern in the resulting backscatter. Small zooplankton can be studied using a high-frequency echosounder when the number of individuals is sufficient, and the zooplankton bodies present sufficient impedance contrast against the surrounding medium [36–38]. The zooplanktonic layer refers to a dense aggregation of zooplankton within the ocean water column, detected acoustically [39, 40]. These zooplanktonic layers typically contain several species of different sizes with vastly different acoustic properties [41]. Stanton et al. [42] classified zooplanktonic organisms into three main scatterer groups based on their material properties: fluid-like(*e*.*g*., euphausiids and copepods), hard elastic-shelled(*e*.*g*., gastropods), and gas-bearing (*e*.*g*., siphonophores). In certain regions, copepods aggregate densely in layers that are sufficiently distinct from other sources of scattering, making them detectable targets for fisheries acoustic devices [37, 41, 43, 44].

Several theoretical scattering models are used to describe the acoustic properties of zooplanktonic organisms [45], such as crustaceans, including copepods, over a wide range of frequencies and organism sizes. Furthermore, various simple models, such as spherical models [46, 47] and cylindrical models [48, 49], are used for different types of organisms to characterize their acoustic scattering properties. Crustaceans are categorized as fluid-like organisms with acoustic characteristics close to those of the surrounding water [50]. Accurately converting acoustic backscatter data to numerical estimates of organism quantities depends on knowledge of the scattering organism size and shape. Fluid-like organisms have increasing frequency response patterns between 38 and 120 kHz, whereas fish with gaseous swim bladders have weakly decreasing frequency response patterns [51]. This difference enables the use of these two frequencies to extract information on fluid-like organisms from acoustic data when other organisms are present (e.g., fish and other zooplankton, i.e., hard elastic-shelled and gas-bearing ones) [51, 52]. Madureira et al. [52] used this dependence of the backscatter on acoustic frequency, namely the difference in the mean volume backscattering strength (ΔMVBS, in dB) at different frequencies, to identify portions of echograms representing krill. Other researchers have used the ΔMVBS method, particularly at the frequencies of 38 and 120 kHz, to discriminate zooplankton species [53–55] and to distinguish zooplankton from fish [51, 56, 57]. Assessing copepods in southern CCLME is crucial for enhancing the management and conservation efforts in these regions.

By estimating copepod abundance and biomass, we can better understand the productivity of the pelagic ecosystem.

To our knowledge, no acoustic characterization of zooplanktonic organisms in the southern part of the Canary Current Large Marine Ecosystem (CCLME) has been performed [55]. In this study, we aim to assess the suitability of bi-frequency acoustic method to estimate copepod density, biomass, and spatial distribution within the southern region of the CCLME during upwelling season. We hypothesized that bi-frequency acoustic methods can reliably estimate copepod (i) density, (ii) biomass and (iii) spatial distribution.

## 2. Materials and methods

### 2.1. Acoustic sampling

**Fig 1:**
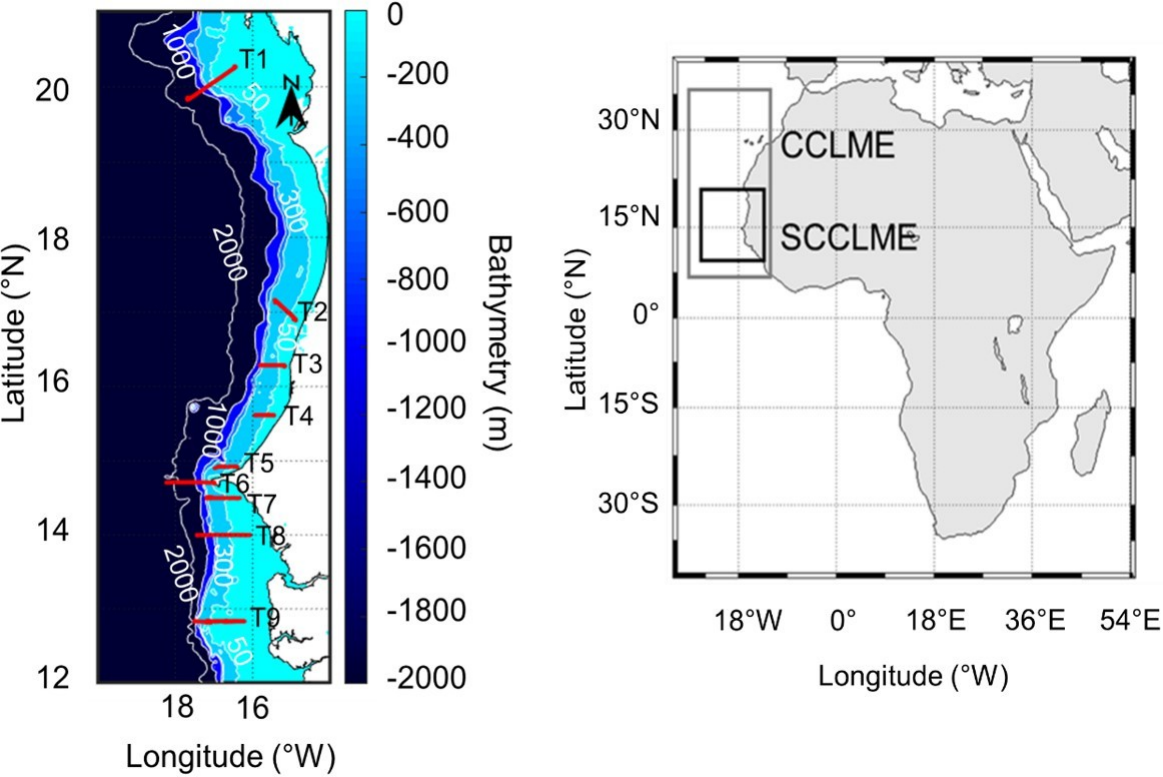
Survey area location off the southern part of the Canary Current Large Marine Ecosystem coast along nine transects (T1-T9) perpendicular to the coast. Bathymetry data from ETOPO1 Global Relief Model (https://www.ncei.noaa.gov/products/etopo-global-relief-model).

The AWA (Ecosystem Approach to the management of fisheries and the marine environment in West African waters) hydroacoustic survey was conducted in the southern part of the CCLME, in Mauritania, Senegal, and The Gambia. The southern CCLME experiences seasonal hydroclimatic variations, alternating between hot, rainy periods and cold, dry conditions corresponding to the upwelling season [58]. The survey was carried out during the upwelling season [59], when the copepod abundance was the highest. In the southern part of the CCLME, copepods are sensitive to environmental changes, making them indicators of ecosystem health [60]. The survey was carried out on-board the R/V Thalassa (Ifremer, France) from February 24 to March 14, 2014, providing a snapshot of the zooplankton distribution during the upwelling season. Acoustic data were collected along nine transects (T1–T9) oriented perpendicular to the coast (Fig 1). Acoustic data were recorded continuously using a Simrad EK60 echosounder connected to 18, 38, 120, 200, and 333 kHz split-beam hull-mounted transducers. This study considered data obtained at 38 and 120 kHz, the most common frequencies used on-board research vessels [44, 51]. The pulse length was set to 1.0 ms for both frequencies. Transmitted powers were set to 2000 and 200 W at 38 and 120 kHz, respectively. Considering the aft draught of the research vessel used, the acoustic near field of the transducer, and the presence of surface air bubbles in the upper part of the water column, data from the surface down to 10 m were not analyzed. The echosounder systems were calibrated using a standard sphere calibration [61] to ensure the reliability of the measurements. To do so, a standard target (a tungsten carbide sphere with known acoustic properties) suspended in the acoustic beam at various angles was used.

### 2.2. Net sampling

Zooplankton samples were collected day and night with a Hydro-Bios MultiNet Midi system at four stations along T3. The MultiNet comprised a 0.25 m^2^ mouth opening, integrated pressure sensor, electronic flow meters, and five nets for depth-stratified sampling. The five nets (mesh size 300 μm) were triggered at different depth intervals: 0–10 m, 10–25 m, 25–50 m, 50–75 m and 75–100 m at station 1; 0–50 m, 50–100 m, 100–200 m, 200–300 m, and 300–600 m at station 2; and 0–100 m, 100–200 m, 200–300 m, 300–600 m, and 600–990 m at station 3.

The MultiNet was deployed and retrieved at a rate of ~1.5 m s^-1^ and was hauled obliquely, and the water flow through the nets was measured. The nets were rinsed after each deployment, and the samples were stored in marked bottles and preserved with 4% buffered formaldehyde in seawater solution [62]. Due to the depth limitations of the acoustic frequencies used (800 m and 250 m for 38 kHz and 120 kHz frequencies, respectively), only MultiNet data from the upper 200 m were considered.

### 2.3. Laboratory analysis of zooplankton data

Zooplankton samples were analyzed using an adaptation of the ZooScan approach [63], an off-the-shelf flatbed scanner (Perfection V750 Pro; Epson), and a scan chamber consisting of a 21 × 29.7-cm (DINA4) glass plate with a plastic frame [64]. First, the formaldehyde was removed from each sample; the sample was gently transferred to a 64-μm sieve and rinsed thoroughly with fresh water. Then, the sample was separated into three size fractions [large (>1000 μm), medium (<1000 μm and >500 μm), and small (<500 μm)] using sieves of respective mesh sizes. Size fractions with very abundant zooplankton were further split with a Motoda splitter so that they could be manipulated on the scanner. The plankton items in the different fractions were distributed and separated on a glass plate on the scanner using tweezers. Eight-bit, 2400-dpi grayscale images (in Tagged Image File Format) were acquired. The scan area was divided in half (*i*.*e*., two images were acquired per frame scanned) to reduce the size of individual images and facilitate the application of ZooProcess [63] to segment the raw images and produce miniature images (vignettes or thumbnails) of single objects. These thumbnails and the image features (*e*.*g*., metadata, x and y dimensions, and area) of all objects were saved, and taxonomic units were assigned automatically using Random Forest classification by EcoTaxa [65], a tool developed for the visual exploration and taxonomic annotation of plankton images. After manual validation of the EcoTaxa outputs, data files for each subsample containing all metadata, measurements (*e*.*g*., body surface area), and taxonomic assignments for objects in the medium and large fractions were uploaded. Only organisms with Equivalent Spherical Radius (ESR) > 0.5 mm were considered in further analyses.

### 2.4. Acoustic data analysis

Integrated echoes were analyzed using Matecho tool [66], an integrative processing software that enables the manual correction and filtering of echograms and echo-integration performance. Echoes were integrated with a -80 dB threshold and at a spatial resolution of three pings per 1 m depth. The Matlab software (version R2018a) was used to extract copepod detections. An approach described by Ballón et al. [67] based on the summation and differences in MVBS (in dB) between frequencies was employed for this purpose.

**Fig 2:**
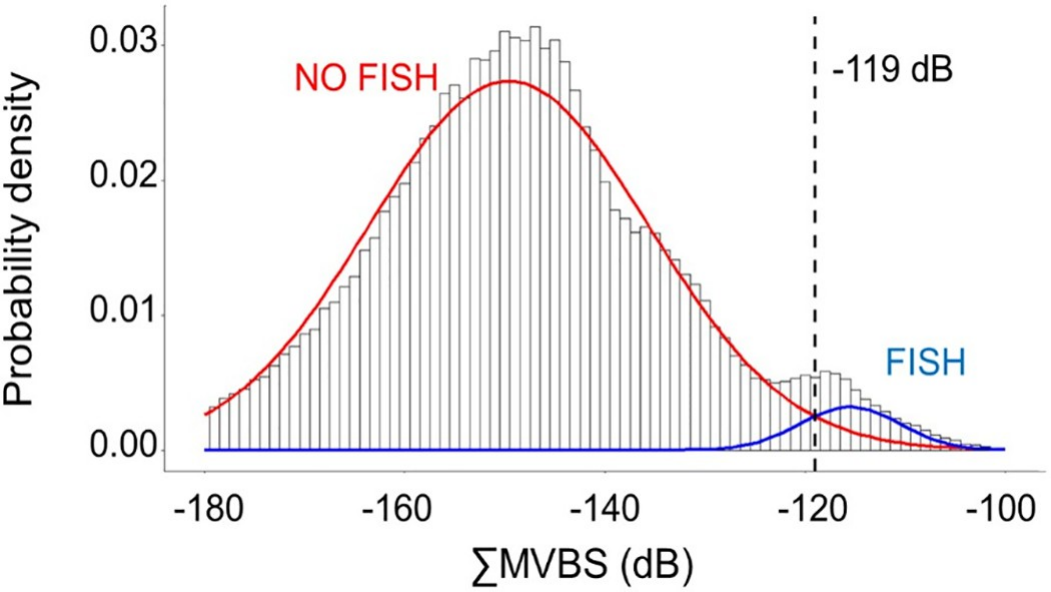
Histogram showing the sum (∑MVBS) of the mean volume backscattering strength (MVBS, in dB) between 120 and 38 kHz frequencies along the sampled transect (T3) with integration cells of three pings per meter. Threshold at -119 dB (dotted line) distinguishing fish (FISH) and no fish (NO FISH) values.

**Fig 3:**
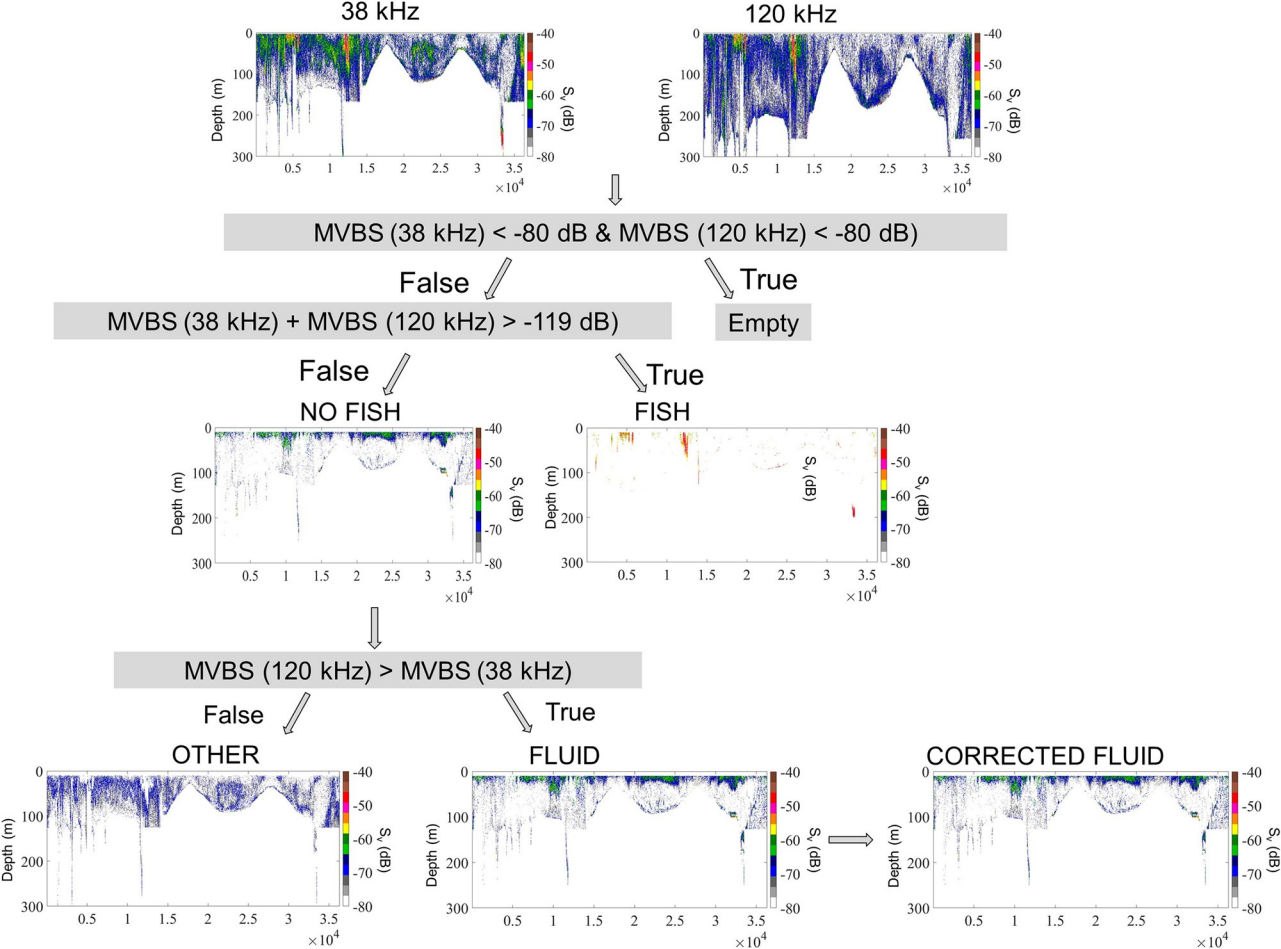
Bi-frequency (38 and 120 kHz) acoustic algorithm for discriminating Fluid-like (“FLUID”) and “Other” (i.e., fish and other scatters) groups using mean volume backscattering strength (MVBS, dB) summation and differences. This method was applied to West African data (2014 AWA survey) to obtain a corrected “Fluid-like” echogram with no fish echo. Adapted from Ballón et al. [67].

The discrimination process involved separating "Fish" and "No fish" groups based on a ∑MVBS threshold of –119 dB, determined using a mixed model of Gaussian distributions (Fig 2). Following this, the "Fluid-like" group was extracted from "No fish" echograms using positive ΔMVBS values (Fig 3). In contrast, targets with negative ΔMVBS values were categorized as "Other." A smoothing process was employed to extract and reintegrate lingering fluid-like echoes in "Fish" echograms to address lingering fluid-like echoes. The discrimination accuracy was further improved by applying an upper threshold of –65 dB to eliminate potential remaining high echoes.

**Fig 4:**
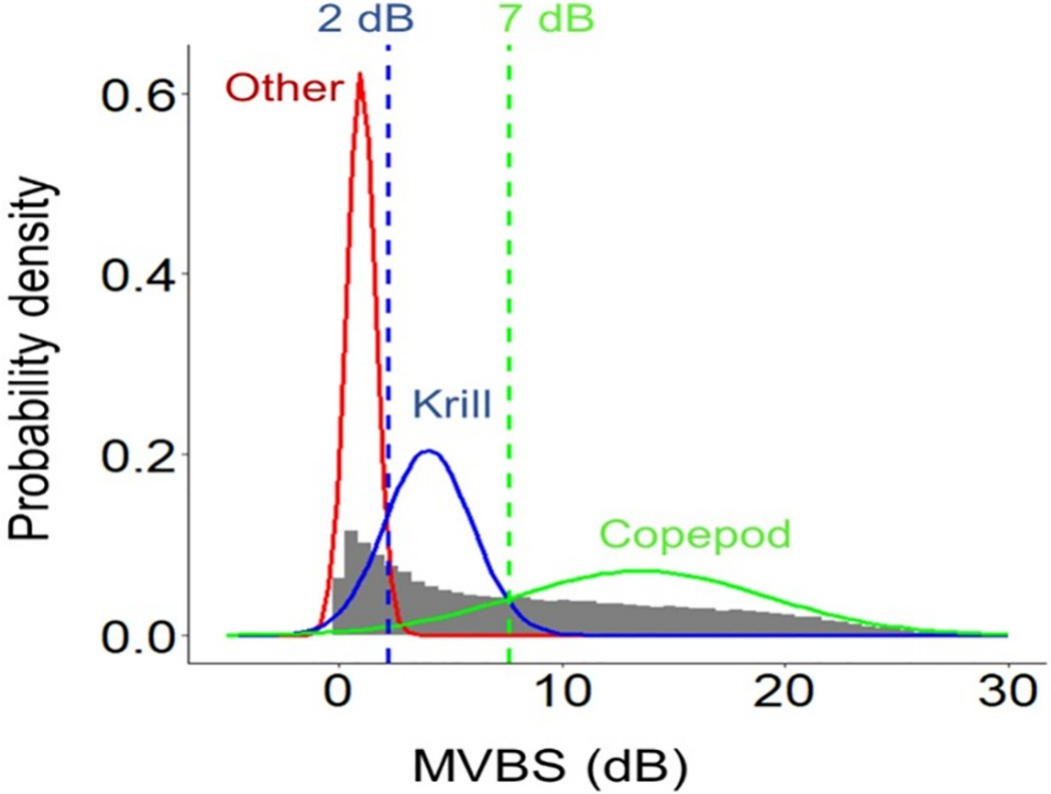
Histogram showing the difference (ΔMVBS) of the mean volume backscattering strength (MVBS, in dB) between 120 and 38 kHz frequencies applied on Fluid-like echograms. The range of 2–7 dB corresponds to krill, while the 7–25 dB range corresponds to copepod.

Although the “Fluid-like” group includes copepods, euphausiids, salps, and siphonophores (without gas), we focused on mesozooplankton (equivalent spherical radius ≥ 0.5 mm), especially copepods. To extract only copepods and remove potential krill echoes that could produce a strong signal, the ΔMVBS histograms of the “Fluid-like” echograms (Fig 4) were analyzed. Three groups of dB intervals corresponding to three biological scatterers were distinguished. Based on these results and on the literature [44, 52, 67], targets for which ΔMVBS < 2 dB were defined as Other, *i*.*e*., fish and other scatters, and the ranges of 2–7 dB and 7–25 dB were taken to correspond to krill and copepods, respectively. To validate the acoustic discrimination method, several comparisons were performed by regressing the following data pairs: MultiNet copepod abundance and biomass against acoustically estimated copepod abundance, MultiNet krill abundance against acoustically estimated krill abundance, and combined MultiNet copepod and krill abundances against corresponding acoustically estimated abundances.

For copepod biomass estimation, the fluid sphere model detailed in Stanton [48] was used to predict ΔMVBS from Fluid-like at 38 and 120 kHz. In this model, the backscattering cross-section (σ_bs_, in m) of any object can be simplified as follows:

$$\sigma_{bs}=\frac{a^{2}{\left(ka\right)}^{4}a_{\prod s}^{2}}{1+\left[4{\left(ka\right)}^{4}a_{\prod s}^{2}\right]/\left(R^{2}\right)}$$ (1)

Where

$$R=\frac{gh-1}{gh+1}$$ (2)

$$\text{for}\;\text{Rayleigh}\;\text{scattering}\left(ka<<1\right):\sigma_{bs}=a^{2}{\left(ka\right)}^{4}{\left(a_{\prod s}\right)}^{2}$$ (3)

$$\text{for}\;\text{geometrical}\;\text{scattering}\left(ka>>1\right):\sigma_{bs}=\frac{1}{4}a^{2}$$ (4)

where

$$a_{\prod }=\frac{1-g}{1+2g}\left(\frac{1-gh^{2}}{3gh^{2}}\right)+\frac{1-g}{1+2g}$$ (5)

“a” is the spherical Radius in meter; “k” is the acoustic wavenumber (k = 2π⁄λ where λ is acoustic wavelength in meter); “g” is the density contrast between the sphere and the surrounding medium, and “h” is the sound speed contrast.

No estimation of “g” and “h” is available for Calanoida, the dominant copepod group on the Senegalese coasts. We used these parameters from the literature: g = 1.02 and h = 1.058 [41]. The use of these parameters for the density and sound speed contrasts is well supported by previous studies on modelling acoustic scattering by fluid-like zooplankton. These values are commonly used when applying the fluid sphere model to represent crustacean groups like copepods and euphausiids. For example, Lavery et al. [41] used the same parameters for modelling the scattering properties of the copepod species *Calanus finmarchicus*. Sakınan et Gücü [37] adopted similar parameters when modelling *Calanus euxinus*. Additionally, these values are within the range typically reported for marine organisms [68], making them reasonable initial assumptions for our calculations. The seawater sound speed was assumed to be 1508 m s^-1^, corresponding to a mean temperature of 14.9°C and a sea salinity of 35.7 PSU.

**Fig 5:**
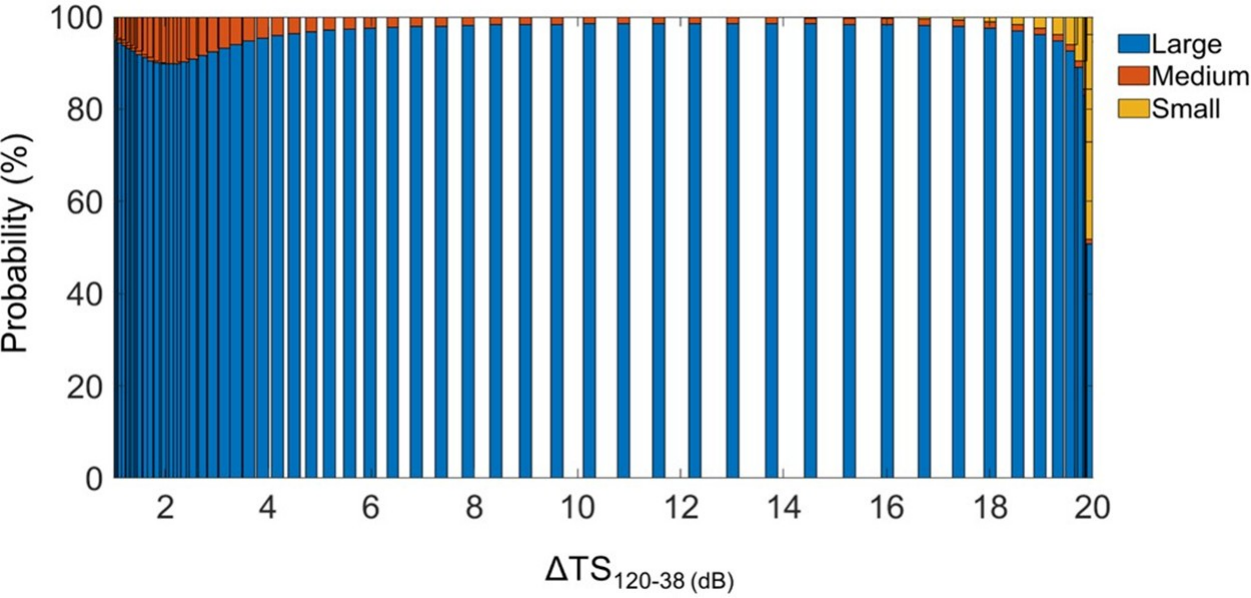
Size class contribution to Target Strength difference. Histogram showing the percentage contribution of different size classes (in Equivalent Spherical Radius) to the target strength (TS, in dB) difference estimated using the fluid sphere model [48] on West African data.

**Fig 6:**
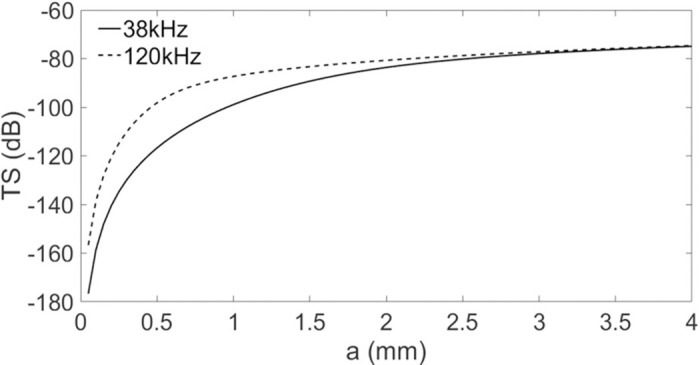
Copepod size vs. Target Strength. Copepod size ‘a’ (in Equivalent Spherical Radius, ESR in mm) vs. detected Target Strength (TS in dB) at frequencies of 38 kHz (full line) and 120 kHz (dotted line).

**Fig 7:**
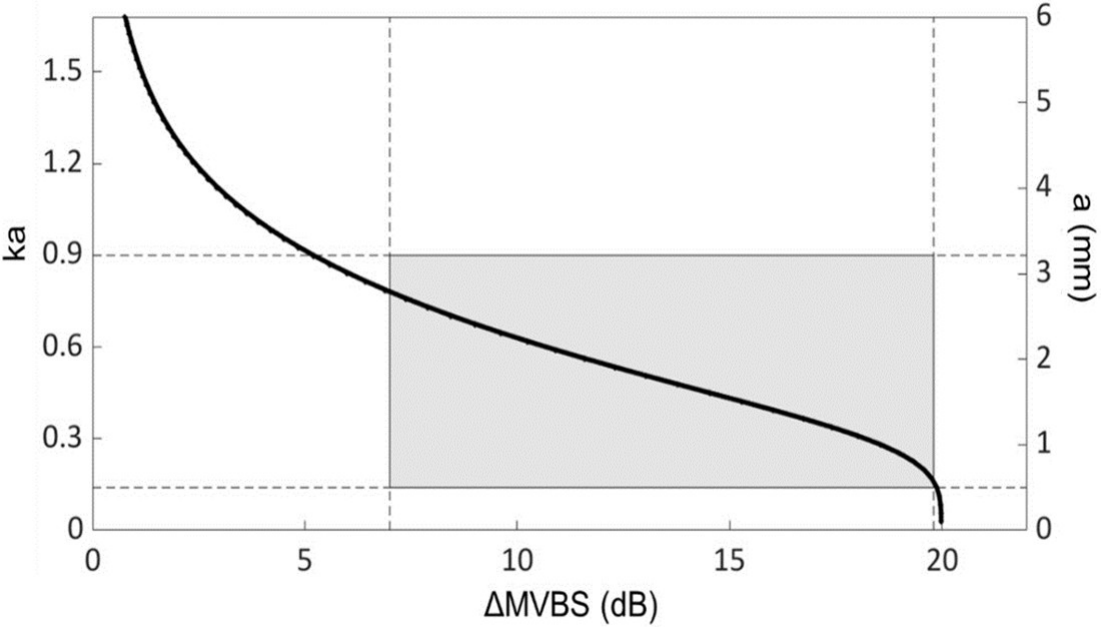
Copepod Target Strength relationship. The relationship between “ka” and the difference in copepod Target Strength (in dB) at 120 and 38 kHz. “k” is the acoustic wavenumber in surrounding water, and “a” is the radius of the animal body in mm. k = 2π fm / c, with c = speed of sound (in m s^-1^), fm = (f_120_*f_38_)^0.5^ (in kHz). The slope of the backscattering difference at low frequencies (small ka) is related to the size of the scatterers. The differences between 7.0 and 19.7 dB correspond to the size range of 0.5 and 3.1mm and the ka range of 0.14 to 0.81. The overlaid shadow area indicates the range at which the high-pass model provides safe radius estimates.

Using the high-pass model,the Target Strength (TS in dB) for sizes (ESR, in mm) ranging from 0.1 to 8.0 mm was calculated. The probability of differences in dB (Δ_TS_) falling within a particular size class (small < 0.5; medium 0.5–1; and large > 1 mm) was computed for Δ_TS_ values ranging from 0 to 20 dB, with 0.5 dB intervals between values (Fig 5). The Large class was highly dominant. Thus, the other classes were not considered in the model. The TS estimated by the model showed a difference at two frequencies between 0 and ~5 mm in size (Fig 6). Beyond this size interval, the TS no longer varies according to the two frequencies. For that reason, considering the size limit (ESR) derived from the MultiNet analyses (S1 Fig) and the MVBS range (7–20 dB) for copepod detection, only the differences comprised between 19.7 and 7.0 dB corresponding to the size range from 0.5 to 3.1 mm ESR, i.e., large class (Fig 7) were used to estimate the copepod density.

The acoustic density of copepod layers was estimated from the Nautical Area Scattering Coefficient (NASC or s_A_, m^2^ nmi^-2^). NASC can be used as a proxy of copepod abundance, assuming that the composition of the layers and the resulting scattering properties of organisms in the layers are homogeneous within each layer over the sampling region [69].

The number of individuals per unit volume (“Nf”, ind m^-3^) can be estimated by:

$$N_{f}=10^{(S_{v\left(f\right)}-\;TS_{f})/10}$$ (6)

where *TS*_*f*_ = 10*log10 (σ_bs_)

S_v_ is the mean acoustic volume backscattering strength (dB); σ_bs_ is the backscattering cross-section (m^2^).

### 2.5. Copepod biomass estimation

#### 2.5.1. Calculation using MultiNet data

To calculate the copepod biomass, the body area of each organism, provided in pixels by ZooScan, was converted to mm^2^ using the following formula:

$$\text{Body}\;\text{area}\left({\text{mm}}^{2}\right)=\text{object}\;\text{area}\left(\text{pixel}\right)*0.0106*0.0106$$ (7)

Where 0.0106 mm is the pixel size (10.6 μm).

Then, body area-to-dry mass conversion factors for subtropical mesozooplankton, based on regression and correlation parameters for subtropical zooplankton [70], were used to calculate the biomass of each scanned mesozooplankton organism [71]. The formula used to calculate the dry mass (DM, in μg) was:

$$\text{DM}=aS^{b}$$ (8)

Where DM is the dry mass (in μg), “a” is the intercept, “S” is the body area in mm^2^, and “b” is the slope.

The DM for each MultiNet station was then calculated as the sum of the individual biomass of all organisms with an ESR > 0.5 mm. For comparison with the hydroacoustic survey data, subsets of only copepod abundance or biomass data were extracted. Other organisms found in the MultiNet samples, such as krill, were not included in biomass estimation in this case, as the inverse method allows only the retrieval of the signal of the most dominant organisms.

#### 2.5.2. Calculation using acoustic data

The numerical density or abundance (N) measurements from each depth were integrated to obtain the total number of mesozooplankton copepods per unit area (N, ind m^-2^) in the epipelagic layer (< 200 m) for each elementary sampling unit of 0.001 nmi. The mean acoustic copepod biomass in the surveyed area (B_m_, g m^-2^) was obtained by multiplying the depth-integrated mean numerical acoustic density (N) by the mean individual dry mass (DM_m_ = 217 μg) obtained from ZooScan. The total biomass (B_t_) in the study area was estimated using a simple interpolation kriging method implemented with the R gstat package [72]. A grid with a cell size of 0.05 degrees was employed for the interpolation, and the formula of total biomass can be expressed as follows:

$${\text{B}}_{t}=\sum_{i=1}^{n}B_{i}*A_{i}$$ (9)

where B_i_ is the estimated biomass of each grid cell, A_i_ is the Area of the grid cell.

All statistical analyses were performed with R software. The spatial auto-correlation in the dataset along transects was assessed using Moran’s Index statistic [73], indicating values below 0.3, suggesting a negligible spatial autocorrelation. Diel transition periods (dusk and dawn) were removed from the analyses to prevent diel vertical migration (DVM) from biasing changes in acoustic density. The transition periods, i.e., around sunset and sunrise, were linked to sun azimuths (i.e., altitudes between –18° and +18°) [74], determined with date, hour, and geospatial position data. Copepod distributions by diel period were represented using box plots, a graphical method for displaying the median, upper and lower quartiles, and minimum and maximum values. To validate the implemented model, the modeled copepod abundance (ind m^-3^) values for all MultiNet station depth strata were compared with the MultiNet-estimated copepod numbers. To compare the abundances obtained from acoustic and MultiNet methods, non-parametric tests were employed. Due to the lack of normal distribution in both the acoustic and MultiNet data, Spearman correlation coefficients and corresponding *p*-values were computed.

## 3. Results

### 3.1. Zooplankton from MultiNets

**Fig 8:**
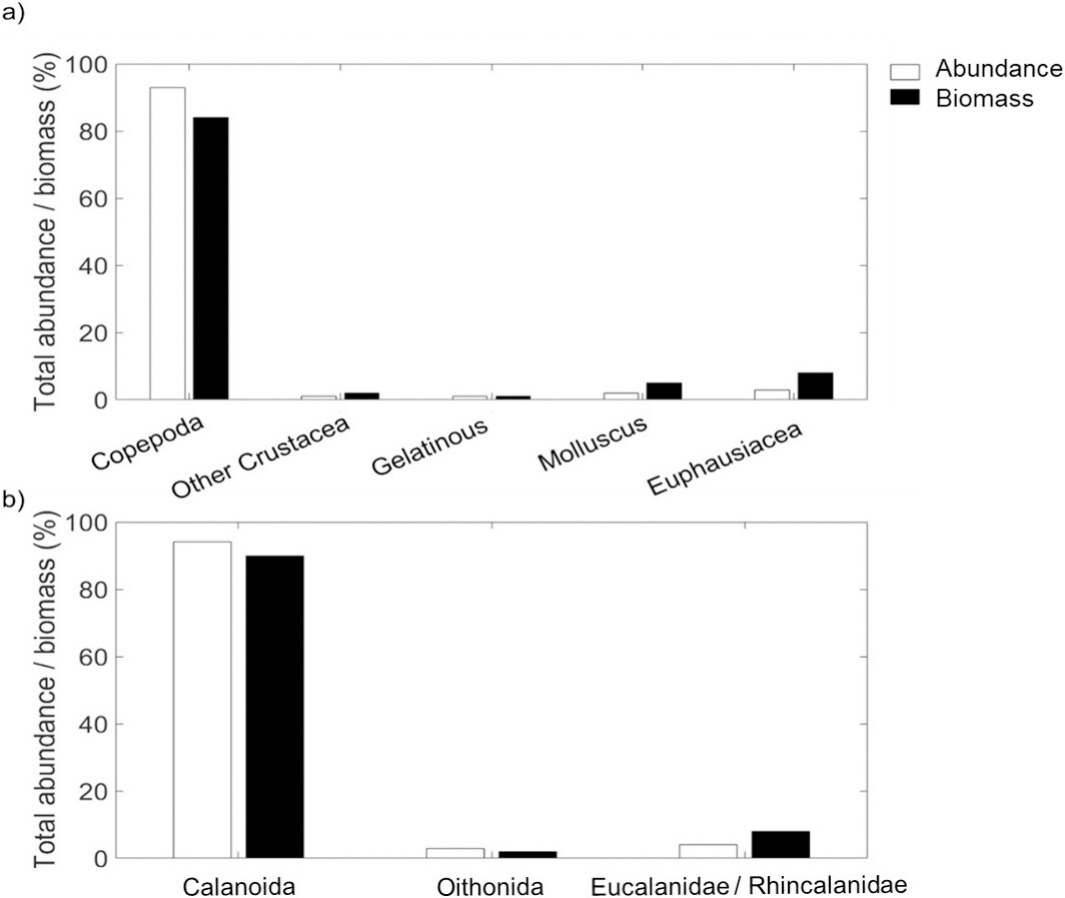
Abundance and biomass percentages. (a) Percentage of total abundance and biomass by zooplankton categories sampled with the Hydrobios MultiNet during the fisheries acoustics AWA sea survey off Senegal; (b) Percentage of total abundance and biomass at copepod level. Only organisms > 0.5 mm ESR are considered.

**Fig 9:**
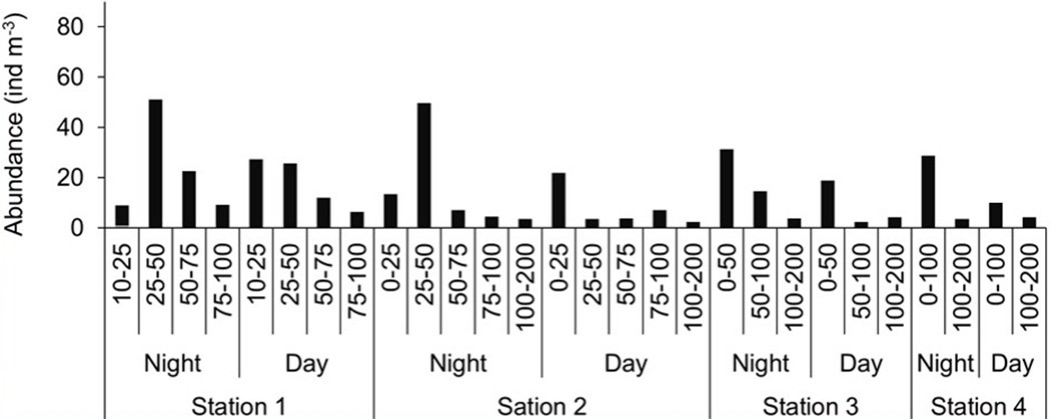
Diel variations in copepod abundance. Daytime and night-time variations of copepod abundance (> 0.5 mm Equivalent Spherical Radius) along the water column at four net stations in various depth layers sampled (from 10-25m to 100–200 m).

Five taxonomic groups were identified from zooplankton analysis (organisms > 0.5 mm ESR): Copepoda, other Crustacea, Gelatinous, Mollusca, and Euphausiacea. Copepods represented 91 and 84% of the total abundance and biomass, respectively. In comparison, the rest accounted for 7 and 16% (Fig 8a). At copepod level, the order Calanoida was the most representative group (94% for total abundance and 90% for total biomass, followed by the suborder Oithonida (3% for total abundance, and 4% for total biomass), and the family Eucalanidae / Rhincalanidae (2% for total abundance, and 4% for total biomass) (Fig 8b). Copepod abundance (> 0.5 mm ESR) varied from the coast to the open sea, with depth and diurnal period. The greatest abundance was observed inshore at station 1 (Fig 9). Copepod abundance was greatest in the upper water column (< 100 m) and greater at night than during the day.

### 3.2. MultiNet and acoustic comparison

**Fig 10:**
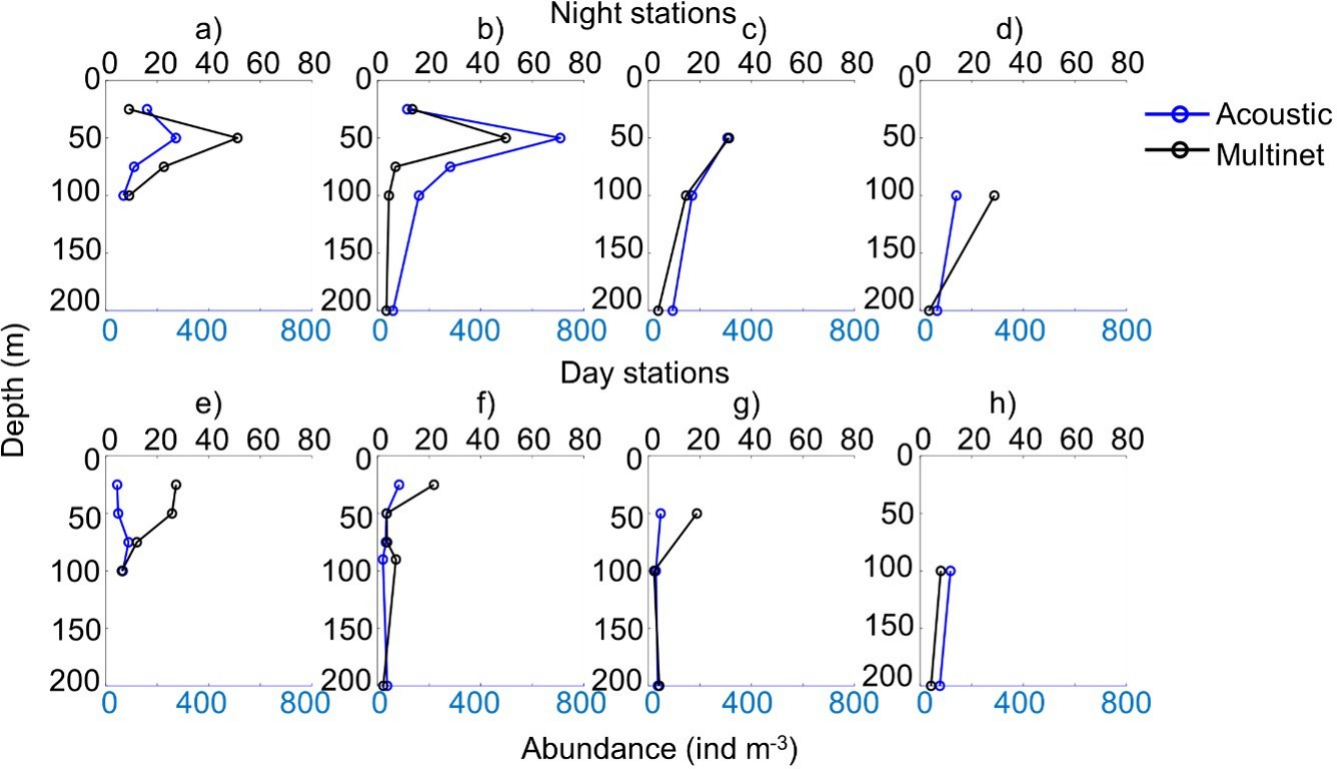
Acoustic vs. MultiNet Abundance. Comparison of copepod acoustic abundances (ind m^-3^) and MultiNet (ind m^-3^; only copepods > 0.5 mm Equivalent Spherical Radius considered) for eight stations per depth strata during a-d) night-time and e-h) daytime stations. Data from survey AWA 2014, West Africa.

**Fig 11:**
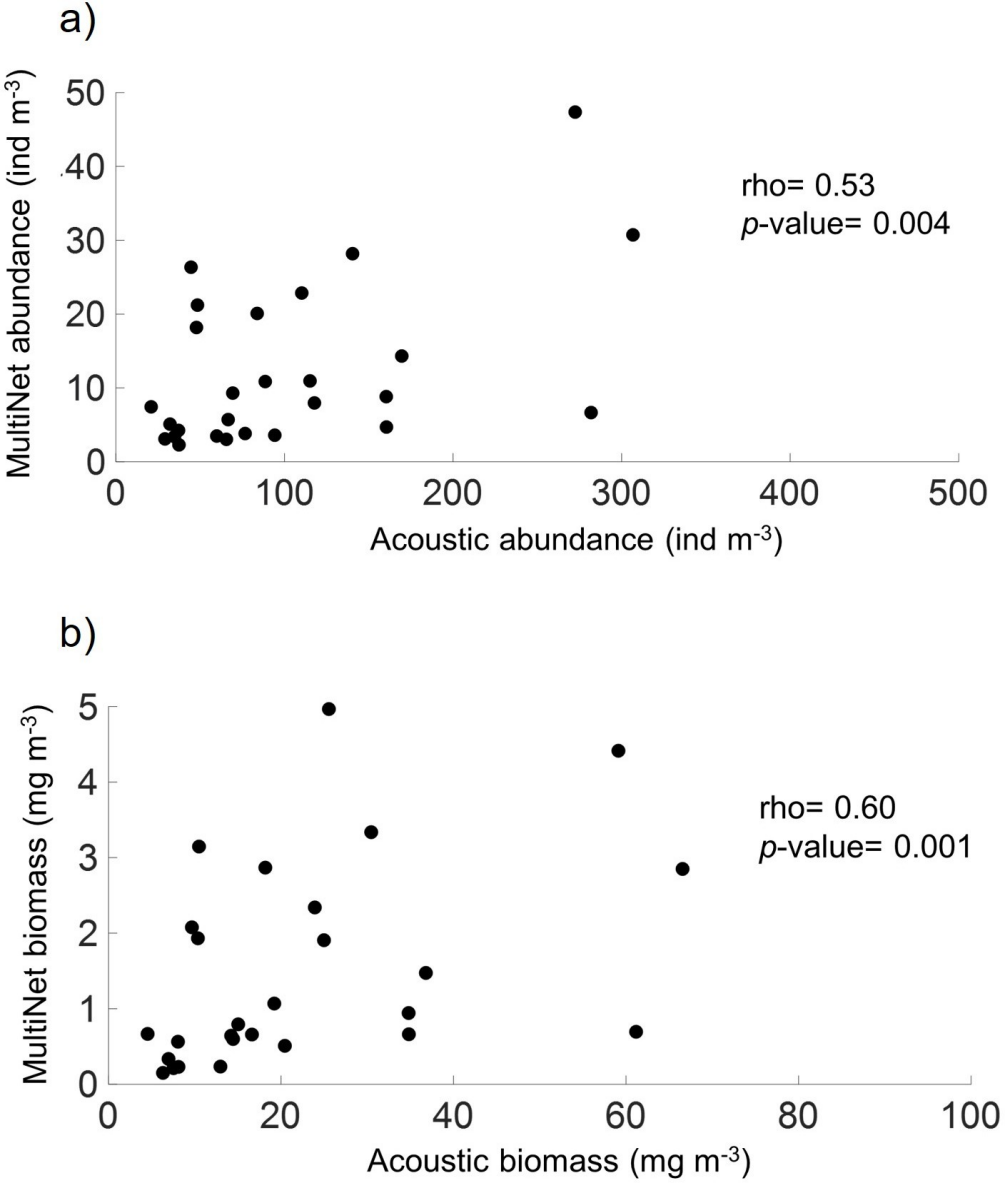
Scatterplots: Copepod acoustic vs. MultiNet estimates. Scatterplots comparison for copepod acoustic and MultiNet estimates (Equivalent Spherical Radius > 0.5 mm) at eight stations: during daytime and night-time (AWA 2014 survey). (a) Abundance (ind m^-3^), (b) biomass (in μg m^-3^). r^2^: Multiple R-squared; Significant *p*-value < 0.03).

For copepod abundance and biomass, acoustic and MultiNet data showed similar daytime and night-time vertical profiles (Fig 10). However, the acoustic estimates were approximately ten times higher compared to the MultiNet. The estimates from both methods were positively correlated for both abundance and biomass (Fig 11). Specifically, the abundance estimates (Fig 11a) showed a Spearman’s correlation coefficient (rho) of 0.53 with a *p*-value of 0.004, indicating a statistically significant positive correlation between the two methods. Similarly, the biomass estimates (Fig 11b) displayed a Spearman’s rho of 0.60 with a *p*-value of 0.001, also suggesting a significant positive correlation. A comparison of the two methods of estimating krill abundance for the purposes of validating the discrimination method also showed consistent diurnal and nocturnal vertical profiles (S2 and S3 Figs), with a significant positive correlation (rho = 0.56, p-value = 0.001).

However, the acoustic method consistently overestimated krill abundances by a factor of approximately 100 compared to the MultiNet estimates. When combining krill and copepods into a single group, discrepancies between the acoustic and MultiNet estimates became apparent, despite similar daytime and night-time vertical profiles (S2 and S3 Figs). The acoustic and MultiNet estimates were not significantly correlated (rho = 0.02, p-value = 0.921), with the acoustic estimates surpassing the MultiNet estimates by a factor of ten (S2 Fig).

### 3.3. Diel variability of copepod biomass

**Fig 12:**
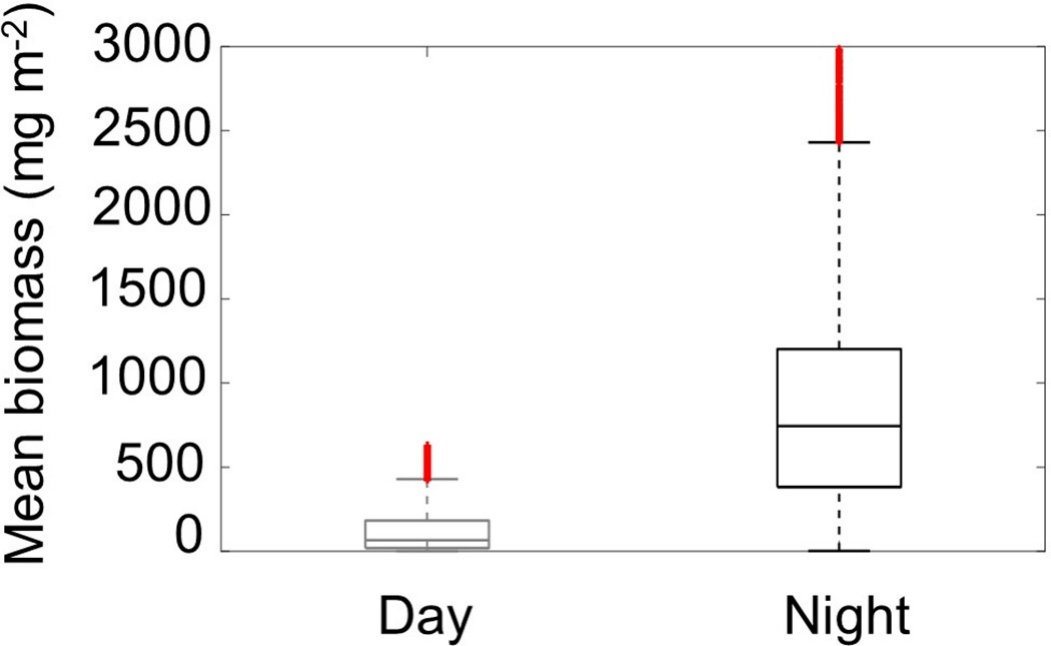
Depth-Integrated biomass variation by diel period. Minimal, maximal, median values and standard errors illustrate the Diel period-dependent variation in depth-integrated mean biomass from hydro acoustics.

The integrated copepod estimated from the MultiNet data (S1 Table) was greater at night than during the day (23.33 *vs* 21.17 mg m^-3^). Depth-integrated acoustic estimates of the copepod biomass exhibited great variation according to the diel cycle (Fig 12). The average TS values for 38 and 120 kHz were -110 and -88 dB re 1 m^-2^, respectively, for a mean mass of 0.217 mg and mean ESR of 0.9 mm. The mean nocturnal epipelagic biomass was estimated to be 850 ± 620 mg m^-2^, almost seven times the estimated diurnal biomass (120 ± 140 mg m^-2^).

### 3.4. Biomass interpolation

**Fig 13:**
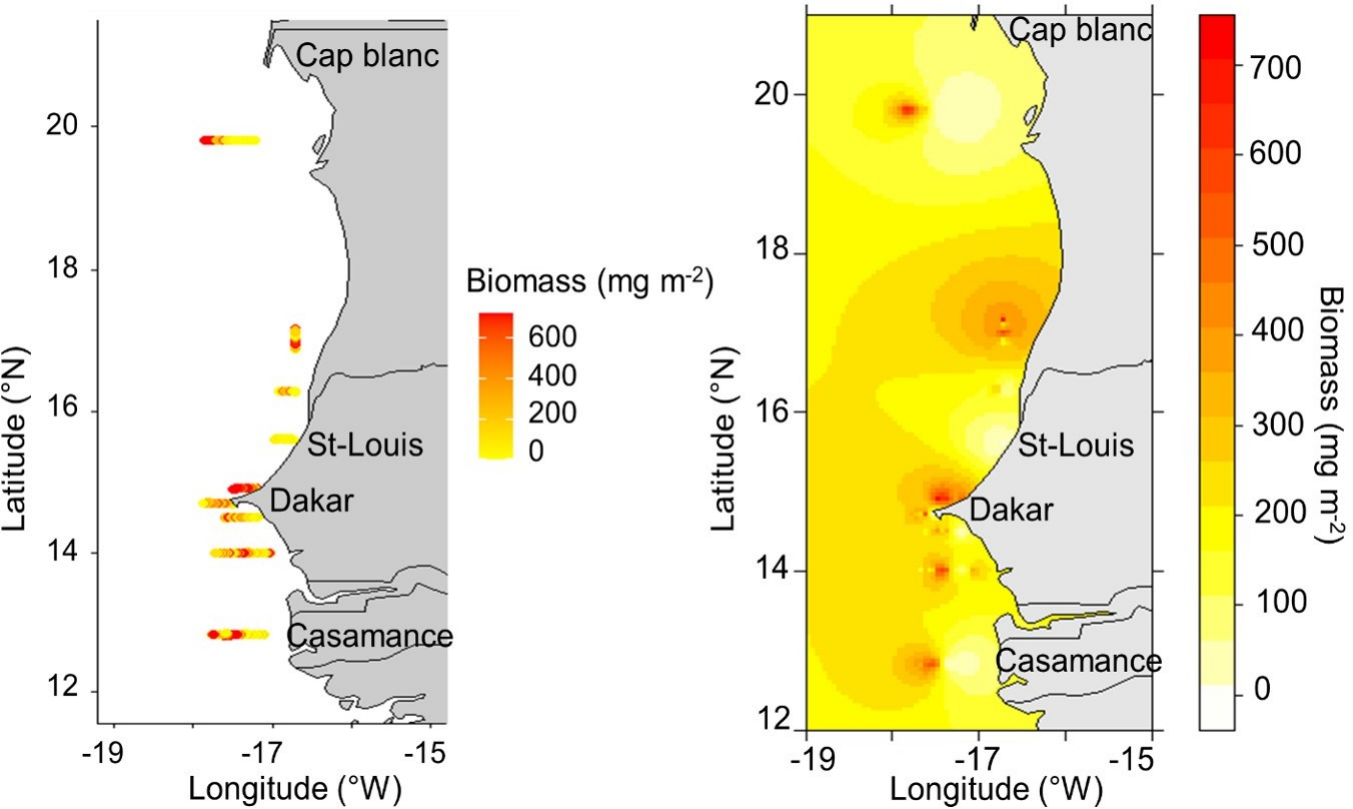
(Left) Observed copepod biomass (mg m^-2^) at net sampling stations along the transect in the southern Canary Current Large Marine Ecosystem (CCLME) based on the March 2014 hydroacoustic AWA sea survey data. (Right) Spatial distribution of interpolated copepod biomass (mg m^-2^) with ordinary kriging in the southern CCLME. The map images were created using the maps package in R, which sources its map data from the Natural Earth project (http://www.naturalearthdata.com/).

The highest copepod biomass (~600 mg m^-2^) was observed just above the Dakar peninsula (Fig 13). High biomass values are also observed in the south of Senegal (towards Casamance), in the north towards Saint-Louis, in Mauritanian waters and off Cap Blanc. The total epipelagic biomass at depths < 200 m was estimated to be 110,123 tons, with a coefficient of variation (CV) of 22.1%.

## 4. Discussion

### 4.1 Methodological considerations and limitations in copepod biomass assessment

#### 4.1.1. Methodological limitations

The results of this study provide the first assessment of the biomass of large copepods in the Northwest African region. It is important to acknowledge several limitations in our study, primarily stemming from the inherent uncertainties associated with the measurement methods.

The temporal coverage of sampling could be extended to better catch the fluctuations in copepod density however [75]. Here we consider the upwelling season, obviously copepod densities may vary depending on the season [76, 77], as do those for other macrozooplankton species [78]. The high abundance of copepods estimated in this study cannot be extrapolated to the hot, rainy season in the CCLME. At the spatial scale, our method allows for fine scale (0.001 nmi) estimates and a wide coverage of the southern CCLME. Nevertheless, zooplankton, including copepods, are known to exhibit patchy distributions that can vary significantly over small spatial scales due to factors such as currents, frontal systems, biological processes like predation and reproduction, as well as environmental variables [79, 80]. The net sampling pattern, while comprehensive, may not have fully captured the patchiness of copepod distribution, which could introduce some biases when using net data to validate the accuracy of acoustic estimates. However, the eight stations used for biological sampling covered a wide range of copepod density variability, ranging from 1.2 to 51 ind m^-3^, which is assumed to encompass the variability within the entire system. Patchy spatial distribution of zooplankton could explain some lower-density estimates derived from net-based sampling.

The spatial distribution and total biomass of copepods in the study area were estimated using ordinary kriging interpolation, a geostatistical method commonly employed for spatial data analysis. Kriging is based on the assumption of spatial autocorrelation, where data points closer together are more likely to have similar values than those farther apart [81]. However, Moran’s Index statistic indicated negligible spatial autocorrelation along the transects, which violates this assumption and could lead to inaccuracies in the interpolated biomass estimates. Another assumption of kriging is stationarity, which implies that the statistical properties (mean and variance) of the variable (in this case, copepod biomass) are constant across the study area [82]. However, zooplankton distributions are known to be patchy and non-stationary, potentially resulting in biased interpolations. Additionally, kriging assumes spatial isotropy, meaning uniform spatial patterns in all directions [82]. Yet, zooplankton distributions can be influenced by directional factors such as currents or frontal systems, introducing anisotropy not addressed by interpolation [83, 84].

On the one hand, the use of the dual-frequency method for the differentiation of fluid-like objects (mainly copepods) from other reflectors at the frequencies of 38 and 120 kHz limited our study to the examination of the epipelagic zone (first 200 m of the water column). On the other hand, the lack of surface acoustic data is also a limitation. Indeed, the first 10 m below the surface was not considered due to the transducer’s hull-mounted depth (6 m), the acoustic Fresnel zone, and surface bubbles, even if copepod is known to occur in significant abundance in the first 10 m [85–87]. In the same way, the research vessel do not cover the shallow part of the shelf (<20 m) and so miss part of the biomass at the coast [88]. Thus, present biomass and abundance estimation may be prone to underestimation. Addressing these limitations could enhance the accuracy of our assessments.

The validation of acoustic discriminations based on net catches is not straightforward because acoustic classifications and catch compositions have different patterns of selectivity [89]. As in our study, fish echoes can mask weak fluid echoes, even when the latter are less abundant in the same sampled volume. Thus, the degree of confidence associated with bi-frequency copepod estimation depends on the type of net used, the acoustic frequencies used, and the species diversity in the environment.

The modelling performed in the present study showed that linking biological compositions or size classes with acoustic data is challenging, especially when the target organisms are not easily detectable because they scatter sound weakly at the frequencies used. The 38 and 120 kHz frequencies are common for zooplankton discrimination [51, 55, 78]. Due to their small size, higher acoustic frequencies (> 30 kHz) are better suited to study these animals [90]. The detectable size of a target is a complex topic that depends on many factors, including the diameter of the animal concerning the acoustic wavelength, orientation, material properties, number of animals per unit volume, the sensitivity of the echosounder, and distance from the transducer [91], and especially on the signal-to-noise ratio (SNR) [92]. A high population density of scatterers can produce a high SNR even if the individual scatterers are small [93].

#### 4.1.2. Encouraging methodological insights

In the present study, the radius size range corresponds to a 7.0–19.5 dB ΔMVBS difference. The dB-differencing method works best when there are large differences (e.g., dB difference > 3 dB) in the groups of species being discriminated against either due to size or because of their composition [94]. Murase et al. [44] reported that ΔMVBS values from a discriminated group can be related directly to size classes, assuming that large differences are related to small sizes and small differences are related to large sizes. In previous studies, mesozooplankton were defined by ΔMVBS values > 12 dB and macrozooplankton/micronekton by values of 2–12 dB [52]. Thus, we can assume that the ΔMVBS range determined in this study encompasses mesozooplankton with an ESR of 0.5 to 3.1 mm. Our results show that TS variation was consistent with Rayleigh scattering of a biological origin, i.e., the TS rapidly increases with increasing frequency. Importantly, the inverse multi-frequency backscatter method enables forecasting dominant scatterers’ size. In many marine ecosystems, copepods are the dominant component but occur in communities of heterogeneous species. Sometimes, their contribution to the overall measured backscattering can be overwhelmed by that of other abundant, large, and/or strongly scattering organisms such as euphausiids, siphonophores, or pteropods [41, 95]. However, Copepoda is the dominant group off Senegalese coasts [80, 96], which in agreement with our results of the MultiNet samples. In addition, the frequency response from the echograms at 38 and 120 kHz confirmed the presence of fluid organisms such as copepods due to the acoustics response, which was higher at 120 than at 38 kHz. In this study, the TS estimates for copepods (0.5 mm< ESR< 2.5mm) ranged from -116 to -80 dB and from -98 to -78 dB at 38 and 120 kHz, respectively (S2 Table). These results are similar to those found in the literature [35, 41, 97] where TS for copepods around 1–3 mm length was reported to be in the range of -100 to -80 dB at 120 kHz, further validating the consistency of our findings with previous studies.

Although the values for copepods obtained from MultiNet data correlated well with those calculated by the model, there was a notable disparity: the acoustic estimates were ten times higher than the MultiNet estimates. One potential explanation for the higher acoustic estimates is the sampling efficiency. Acoustic estimation provides integrated measurements over a larger volume of water, potentially capturing a larger copepod population compared to the targeted depths of the MultiNet sampling [98]. This difference in sampling volume and depth coverage can contribute to the observed discrepancy. Previous studies have reported acoustic estimates that are substantially higher than the MultiNet estimates [37, 67]. For example, Ballón et al. [67] found acoustic estimates to be ten times higher than the MultiNet estimates and attributed this large difference to the avoidance of the net by euphausiids. However, while net avoidance has been reported for larger zooplankton like euphausiids, it is less likely to be a significant issue for smaller copepods in the 1–3 mm length range when using a MultiNet. Other authors [37] discriminated copepods (*Calanus euxinus*) using the Born approximation model and noted that acoustic estimates of copepod abundance were 13% higher than net estimates. TS variability is another factor that could contribute to the differences between the two methods. Copepods exhibit natural variations in size, orientation, and composition, which affect their acoustic reflectivity [99]. If the modelling assumptions do not adequately account for these variations, the acoustic method can overestimate copepod abundance [100].

Furthermore, TS models can be sensitive to the acoustic properties of the materials, i.e. the contrasts in sound speed (h) and density (g) between the animal and the surrounding seawater [99]. Parameters used in the model, such as g and h, were taken from the literature, and their real values for the precise species of this study are unknown. In our study, the high-pass model enabled copepod assessment that agreed well with the large copepod abundance estimated from the MultiNets samples, as evidenced by a significant correlation. However, when comparing combined krill and copepod multinet data with corresponding acoustic data, there was no significant correlation (S2 and S3 Figs). We can, therefore, deduce that the high-pass model is suitable for detecting copepod mesozooplankton in the southern part of the CCLME.

The copepod biomass estimation method described in this paper can be applied in other ecosystems where copepods dominate the zooplankton community with large size species. It provides more detailed information than extraction from global estimates [101] at the world level.

### 4.2. Copepod ecology and behavior

The copepod biomass in southern CCLME exhibited an approximately 7-fold increase during night-time compared to daytime (based on acoustic data), attributed to the DVM phenomenon. Many species of zooplankton and micronekton perform DVM, usually characterized by an ascent towards the surface during the night to feed and a descent during the day to avoid predation [39, 59]. This behaviour is common to the Large Marine Ecosystems (LMEs) worldwide of Eastern Boundary Upwelling (EBU; [102]), e.g. Humboldt, Benguela, and California Currents [103–105]. Furthermore, this study is limited to the epipelagic zone (vs 120 kHz range efficiency), whereas the organisms can migrate into the mesopelagic zone. This explains why the density observed is higher at night than during the day. We recommend exploring the interest in evaluating copepod biomass using exclusively night-time data.

This study investigated the spatial distribution of copepod biomass through modelling and interpolation methods and identified areas of high copepod biomass concentration within the southern CCLME. The highest copepod density was found near the Dakar peninsula (Kayar), with other high values near Casamance (southern, Senegal), Saint-Louis (northern Senegal), and off Cap Blanc (Mauritania). Our findings align with a prior net assessment survey conducted in Senegal during the same season [106]. Our results are in agreement with high zooplankton densities in the upper water column for similar areas off the Senegalese coast [80]. Zooplankton populations appeared to thrive in the northern area (north of Dakar peninsula) due to the influence of coastal upwelling waters. In contrast, in the southern region (south of Dakar peninsula), they benefited from the influence of the less saline coastal waters originating from The Gambia and Casamance rivers [80] as well as the impact of the seasonal upwelling in southern Senegal [58].

This study estimated the acoustic copepod biomass to be 110,123 tons, 120–850 mg m^-2^ (DM) in the southern CCLME at the time of the survey. It is seldom to find specific large copepod biomass estimate in the literature. In the southern Benguela Current, the highest levels of copepod biomass were found to be 3.1 g C m^-2^, which is equivalent to approximately 7750 mg m^-2^ in DM, assuming carbon content values are 40% of dry mass [107]. In the Humboldt Current System, mean copepod biomass ranged from 444 to 1138 mg C m^-2^ *i*.*e*. 1110 to 2845 mg m^-2^ (DM). When comparing these values to mesozooplankton wet mass estimates from the main upwelling systems [67], our estimates are obviously lower [Canary Current values ranging from 3,600 to 56,900 mg m^-2^; in the California, Humboldt, and Benguela LMEs around 8,000, 17,900, and 21,400 mg m^-2^, respectively]. Such difference are caused by the varying species composition of mesozooplankton in these upwelling systems. In our study the large fraction of zooplankton in biological sampling represents 18% of the total (S4 Fig) *vs* the small and medium fractions. Moreover, copepods are assumed to represent 20% of total zooplankton in our study area [77]. And last our results are expressed in DM rather than wet biomass (~90% of biomass; [108]).

The copepod biomass estimated in this study is of relevant interest for fishery resources management. Such estimation will allow to improve the ecosystemic approaches of fisheries in the southern part of the CCLME. These fish species heavily rely on mesozooplankton, including copepods, as a primary food source. Fréon et al. [79] emphasized the role of coastal upwelling systems in enhancing nutrient enrichment and thus promoting copepod productivity. Upwelling increases the availability of nutrients and leads to greater primary productivity, which supports larger copepod biomasses [109–111]. Climate change is expected to alter the intensity and timing of upwelling events in the CCLME [112, 113], which could have significant consequences for the copepod community and so the pelagic food web. Cropper et al. [114] projected that under future climate scenarios, the intensity of upwelling-favorable winds in the CCLME could increase, leading to enhanced upwelling. While increased upwelling could potentially support higher primary productivity and copepod biomass [115]. Moreover, changes in the size structure of the copepod community, with a shift towards smaller species, could affect the overall productivity of the pelagic food web [116].

## 5. Conclusions

This work represents an essential step in the remote discrimination of copepods, a widely dominant mesozooplankton group in the southern CCLME. Further net sampling will enhance the accuracy of the estimation. The bi-frequency approach paves the way for biomass assessment and the revisiting of CCLME fisheries acoustics time series obtained over three decades to scrutinize changes in the ecosystem’s functioning and organization. We confirmed the utility of using 38 kHz for large copepod detection. The combined analysis of 38 and 120-kHz echosounder data, the ∑MVBS, and the ΔMVBS enabled the identification of fish echoes and their removal from echograms. This study showed that the high-pass bi-frequency method provides a complementary approach that is less time-consuming than traditional sampling methods and results that are not sensitive to net avoidance. However, further investigations are needed to understand the large differences between the assessments obtained by both methods, including the use of more complex models such as the DWBA ones [117] that account for the anatomical complexities of weak scatterers. To quantify zooplankton with smaller ESR (<0.5 mm), an echosounder system with higher frequencies would be required [41]. The acoustic assessment of the larger fraction of the copepod biomass underscores the significant productivity of the southern CCLME. Looking ahead, reanalyzing several decades’ worth of archived bi-frequency acoustic data could provide unprecedented insights into long-term changes in the base of the Northwest African marine food webs *vs* hydroclimatic fluctuations and trends.

## Supporting information

S1 Fig Equivalent Spherical Radius (ESR) distribution of copepods measured from the MultiNet samples. The vertical red line depicts the mean value.
(TIF)

S2 Fig Comparison of Krill (Top) and combined group, i.e. krill & copepod (down) acoustic abundances (Acous, in ind m^-3^) and MultiNet (Multi in ind m^-3^) for eight stations per depth strata; a)-d): daytime; e)-h): night-time stations. Data survey AWA 2014, West Africa.
(TIF)

S3 Fig Scatterplots comparison for acoustic and MultiNet estimates (ESR > 0.5 mm) for (a) krill abundance. Spearman correlation coefficient (ρ = 0.4, *p*-value = 0.01); (b) All (krill + copepod). Spearman correlation coefficient (ρ = 0.5, *p*-value = 0.01).
(TIF)

S4 Fig Percentage of abundance per size class (small, medium and large). (TIF)

S1 Table Metadata for MultiNet analysis of Hydrobios MultiNet nets (1 to 5) per station for the two size fractions (medium 500–1000 and large > 1000 μm) from Zooscan analysis during the AWA sea survey. (DOCX)

S2 Table Size (‘a’ mm, in equivalent spherical radius, ESR) *vs* Target Strength (TS, in dB) at 38 and 120 kHz emitting frequencies using our high-pass model. (DOCX)
