# Supplementary figures and images for: Estimating the copepod biomass in the North West African upwelling system using a bi-frequency acoustic approach
Source: PLoS One. 2024 Sep 6;19(9):e0308083. doi: 10.1371/journal.pone.0308083 (PMC11379317; doi:10.1371/journal.pone.0308083)

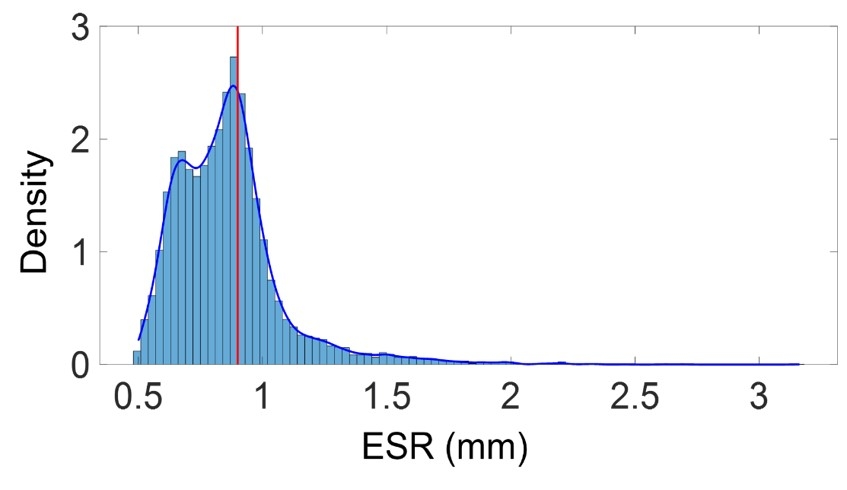

Supplement: S1 Fig — The vertical red line depicts the mean value. (TIF) [file pone.0308083.s001.tif]

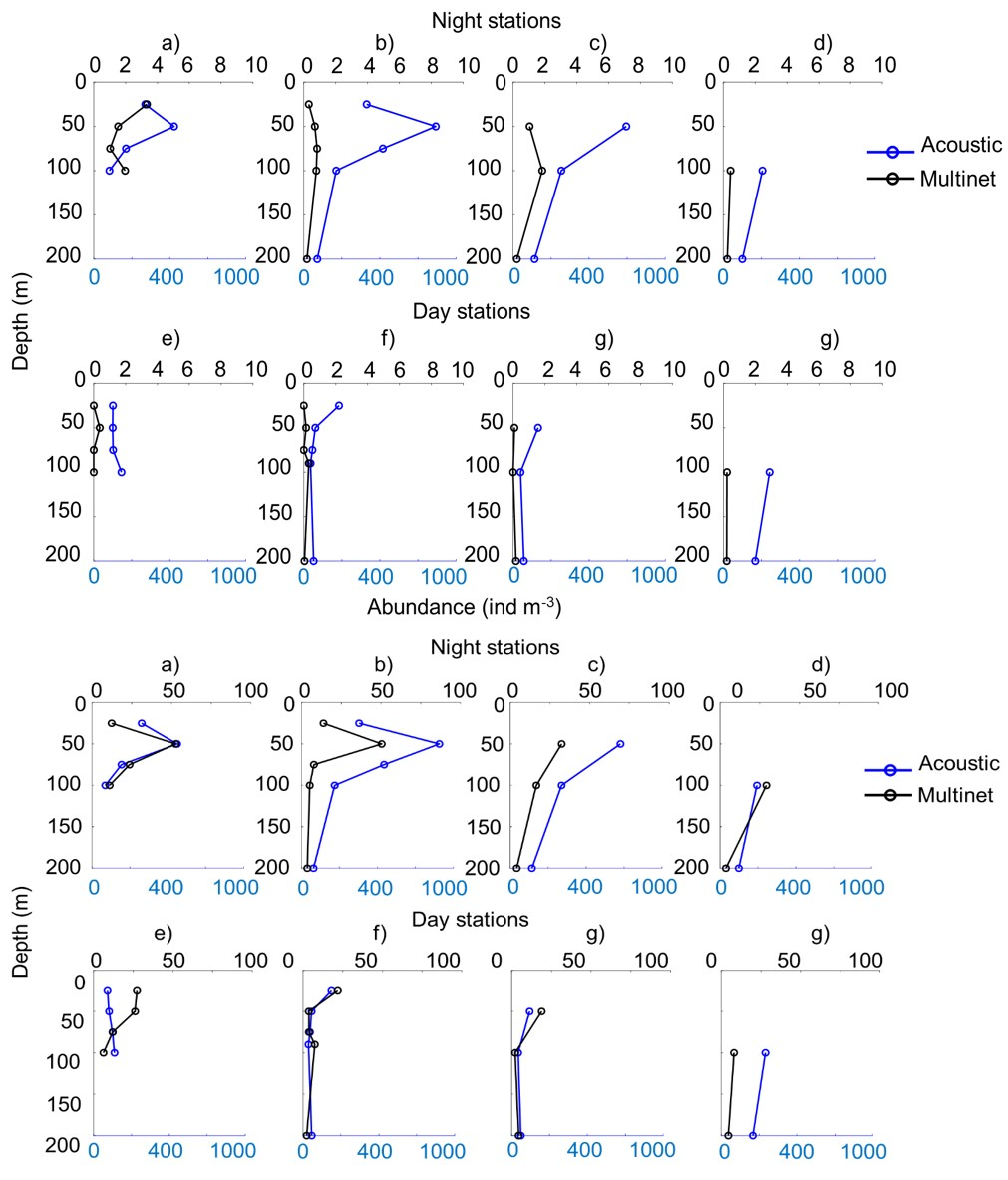

Supplement: S2 Fig — Comparison of Krill (Top) and combined group, i.e. krill & copepod (down) acoustic abundances (Acous, in ind m-3) and MultiNet (Multi in ind m-3) for eight stations per depth strata; a)-d): daytime; e)-h): night-time stations. Data survey AWA 2014, West Africa. (TIF) [file pone.0308083.s002.tif]

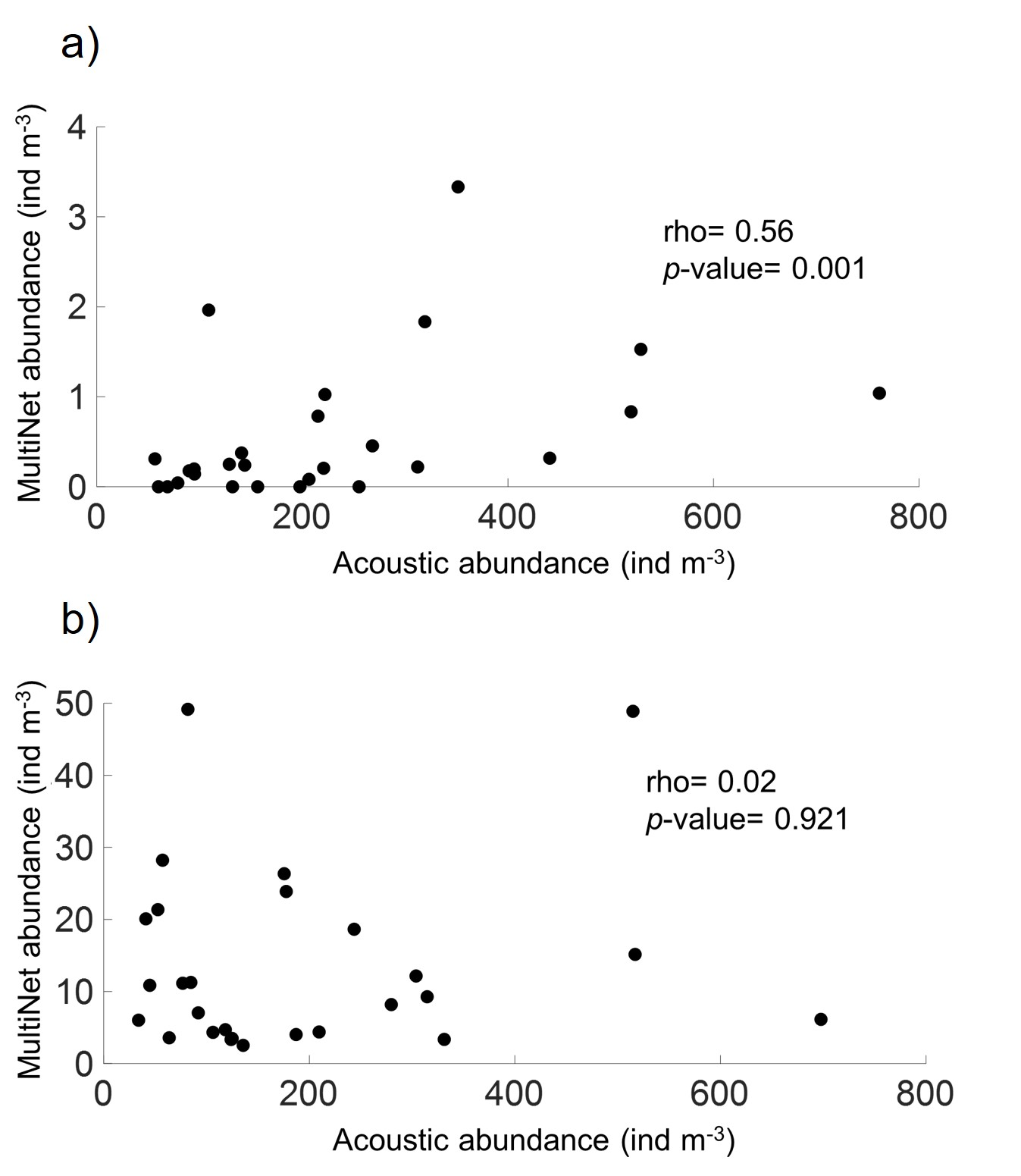

Supplement: S3 Fig — Scatterplots comparison for acoustic and MultiNet estimates (ESR > 0.5 mm) for (a) krill abundance. Spearman correlation coefficient (ρ = 0.4, p-value = 0.01); (b) All (krill + copepod). Spearman correlation coefficient (ρ = 0.5, p-value = 0.01). (TIF) [file pone.0308083.s003.tif]

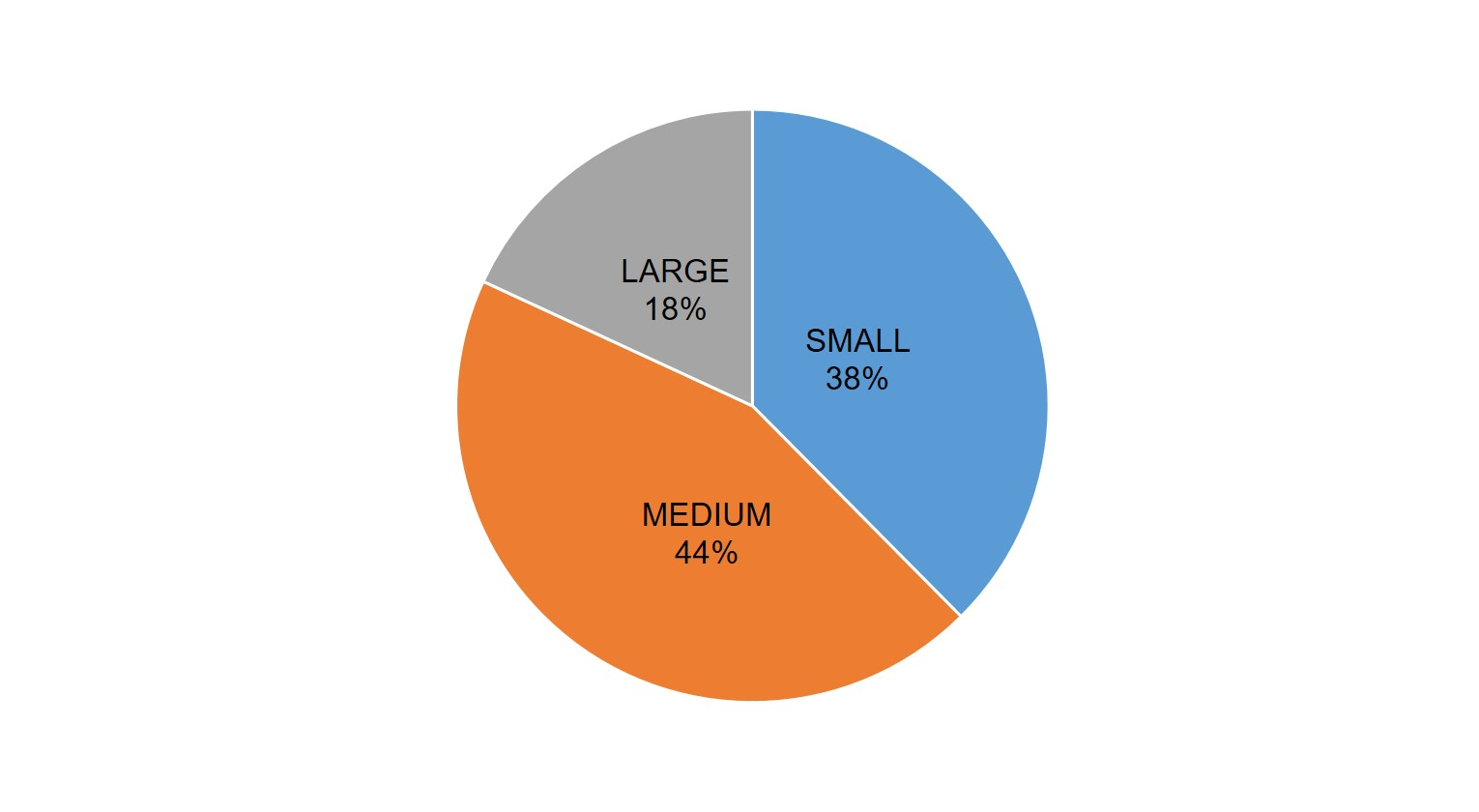

Supplement: S4 Fig — (TIF) [file pone.0308083.s004.tif]
