# Supplementary material for: Estimating the copepod biomass in the North West African upwelling system using a bi-frequency acoustic approach
Source: PLoS One. 2024 Sep 6;19(9):e0308083. doi: 10.1371/journal.pone.0308083 (PMC11379317; doi:10.1371/journal.pone.0308083)
Supplement: S1 Table — (DOCX) [file pone.0308083.s005.docx]

**Diogoul et al. Supplementary material : Tables**

Table S.1: Metadata for MultiNet analysis of Hydrobios MultiNet nets (1 to 5) per station for the two size fractions (medium 500 - 1000 and large > 1000 μm) from Zooscan analysis during the AWA sea survey.

| Station | Net | Date | Start time  (hh:mn:ss) | End time  (hh:mn:ss) | Longitude (°W) | Latitude (°N) | Minimal depth (m) | Maximal depth (m) | Sampled volume (m^-3^) | Medium Fraction | | Large Fraction | |
| --- | --- | --- | --- | --- | --- | --- | --- | --- | --- | --- | --- | --- | --- |
|  |  |  |  |  |  |  |  |  |  | Split ratio | Biomass (µg m^-3^) | Split ratio | Biomass (µg m^-3^) |
| Station1 (Night) | n1 | 03/03/2014 | 01:21:30 | 01:27:23 | -16.95 | 16.3 | 75 | 100 | 9.675 | 1 | 364.73 | 1 | 1965.77 |
|  | n2 |  |  |  |  |  | 50 | 75 | 6.825 | 1/2 | 1190.03 | 1 | 4759.31 |
|  | n3 |  |  |  |  |  | 25 | 50 | 7.2 | 1/8 | 651.16 | 1 | 9217.69 |
|  | n4 |  |  |  |  |  | 10 | 25 | 5.1 | 1 | 1332.03 | 1 | 8345.89 |
| Station2 (Night) | n1 | 03/03/2014 | 02:13:25 | 02:25:27 | -16.84 | 16.28 | 100 | 200 | 30.375 | 1/2 | 106.76 | 1 | 349.08 |
|  | n2 |  |  |  |  |  | 75 | 100 | 6.375 | 1 | 641.98 | 1 | 1655.05 |
|  | n3 |  |  |  |  |  | 50 | 75 | 7.2 | 1/2 | 580.01 | 1 | 1140.04 |
|  | n4 |  |  |  |  |  | 25 | 50 | 7.2 | 1/8 | 1798.05 | 1 | 4839.31 |
|  | n5 |  |  |  |  |  | 0 | 25 | 6.3 | 1/16 | 621.31 | 1 | 5757.26 |
| Station3 (Night) | n3 | 03/03/2014 | 03:24:51 | 03:39:33 | -17.24 | 14.5 | 100 | 200 | 24.975 | 1/2 | 102.03 | 1 | 630.17 |
|  | n4 |  |  |  |  |  | 50 | 100 | 14.175 | 1/2 | 544.54 | 1 | 10707.14 |
|  | n5 |  |  |  |  |  | 0 | 50 | 10.575 | 1/16 | 974.90 | 1 | 8048.43 |
| Station4 (Night) | n4 | 03/03/2014 | 05:04:06 | 05:22:54 | -16.88 | 16.3 | 100 | 200 | 24 | 1/2 | 203.42 | 1 | 1672.43 |
|  | n5 |  |  |  |  |  | 0 | 100 | 26.4 | 1/32 | 199.63 | 1 | 6554.14 |
| Station1 (Day) | n1 | 03/03/2014 | 15:07:37 | 15:13:57 | -16.95 | 16.3 | 75 | 80 | 3.675 | 1 | 347.30 | 1 | 599.63 |
|  | n2 |  |  |  |  |  | 50 | 75 | 4.05 | 1 | 280.20 | 1 | 1068.90 |
|  | n3 |  |  |  |  |  | 25 | 50 | 5.325 | 1/4 | 1953.64 | 1 | 3195.18 |
|  | n4 |  |  |  |  |  | 10 | 25 | 3.15 | 1/4 | 2151.32 | 1 | 2077.93 |
| Station2 (Day) | n1 | 03/03/2014 | 16:28:17 | 16:50:07 | -16.84 | 16.28 | 100 | 200 | 23.55 | 1/2 | 174.10 | 1 | 247.10 |
|  | n2 |  |  |  |  |  | 75 | 100 | 3.225 | 1 | 310.91 | 1 | 703.11 |
|  | n3 |  |  |  |  |  | 50 | 75 | 5.1 | 1 | 179.50 | 1 | 335.18 |
|  | n4 |  |  |  |  |  | 25 | 50 | 7.05 | 1 | 120.89 | 1 | 386.20 |
|  | n5 |  |  |  |  |  | 0 | 25 | 5.625 | 1/8 | 2104.76 | 1 | 2867.88 |
| Station3 (Day) | n3 | 03/03/2014 | 16:37:01 | 16:50:07 | -17.24 | 14.5 | 100 | 200 | 22.575 | 1/4 | 191.42 | 1 | 2364.14 |
|  | n4 |  |  |  |  |  | 50 | 100 | 11.625 | 1 | 135.65 | 1 | 150.81 |
|  | n5 |  |  |  |  |  | 0 | 50 | 12.15 | 1/8 | 472.48 | 1 | 2336.01 |
| Station4 (Day) | n4 | 03/03/2014 | 17:42:40 | 17:59:00 | -16.88 | 16.3 | 100 | 200 | 24.3 | 1/4 | 184.55 | 1 | 1758.92 |
|  | n5 |  |  |  |  |  | 0 | 100 | 27.3 | 1/64 | 360.54 | 1 | 5689.71 |
