# Supplementary material for: Estimating the copepod biomass in the North West African upwelling system using a bi-frequency acoustic approach
Source: PLoS One. 2024 Sep 6;19(9):e0308083. doi: 10.1371/journal.pone.0308083 (PMC11379317; doi:10.1371/journal.pone.0308083)
Supplement: S2 Table — (DOCX) [file pone.0308083.s006.docx]

**Diogoul et al. Supplementary material : Tables**

Table S2: Size (‘a’ mm, in equivalent spherical radius, ESR) *vs* Target Strength (TS, in dB) at 38 and 120 kHz emitting frequencies using our high-pass model.

| ESR | TS_38_ | TS_120_ |
| --- | --- | --- |
| 0.50 | -116.69 | -98.15 |
| 0.55 | -114.22 | -96.21 |
| 0.60 | -111.96 | -94.56 |
| 0.65 | -109.89 | -93.16 |
| 0.70 | -107.97 | -91.96 |
| 0.75 | -106.20 | -90.93 |
| 0.80 | -104.54 | -90.02 |
| 0.85 | -102.99 | -89.23 |
| 0.90 | -101.54 | -88.52 |
| 0.95 | -100.17 | -87.89 |
| 1.00 | -98.88 | -87.31 |
| 1.05 | -97.67 | -86.78 |
| 1.10 | -96.52 | -86.29 |
| 1.15 | -95.43 | -85.84 |
| 1.20 | -94.40 | -85.41 |
| 1.25 | -93.43 | -85.01 |
| 1.30 | -92.51 | -84.63 |
| 1.35 | -91.63 | -84.27 |
| 1.40 | -90.80 | -83.93 |
| 1.45 | -90.01 | -83.60 |
| 1.50 | -89.27 | -83.29 |
| 1.55 | -88.56 | -82.99 |
| 1.60 | -87.89 | -82.70 |
| 1.65 | -87.25 | -82.42 |
| 1.70 | -86.65 | -82.15 |
| 1.75 | -86.07 | -81.89 |
| 1.80 | -85.53 | -81.64 |
| 1.85 | -85.01 | -81.39 |
| 1.90 | -84.52 | -81.15 |
| 1.95 | -84.06 | -80.92 |
| 2.00 | -83.61 | -80.70 |
| 2.05 | -83.19 | -80.48 |
| 2.10 | -82.79 | -80.27 |
| 2.15 | -82.41 | -80.06 |
| 2.20 | -82.05 | -79.86 |
| 2.25 | -81.70 | -79.66 |
| 2.30 | -81.37 | -79.47 |
| 2.35 | -81.05 | -79.28 |
| 2.40 | -80.75 | -79.09 |
| 2.45 | -80.46 | -78.91 |
| 2.50 | -80.18 | -78.74 |
